# Clinician identification of youth abusing over-the-counter products for weight control in a large U.S. integrated health system

**DOI:** 10.1186/2050-2974-1-40

**Published:** 2013-10-21

**Authors:** S Bryn Austin, Robert B Penfold, Ron L Johnson, Jess Haines, Sara Forman

**Affiliations:** 1 Division of Adolescent and Young Adult Medicine, Boston Children’s Hospital, 333 Longwood Ave., #634, Boston, MA 02115, USA; 2 Department of Social and Behavioral Sciences, Harvard School of Public Health, Boston, MA, USA; 3 Group Health Research Institute, Seattle, WA, USA; 4 Department of Health Services Research, University of Washington, School of Public Health, Seattle, WA, USA; 5 Department of Family Relations and Applied Nutrition, University of Guelph, Guelph, Canada

**Keywords:** Adolescence, Diet pills, Diuretics, Laxatives, Eating disorder, Electronic medical record

## Abstract

**Background:**

Abuse of over-the-counter (OTC) products, such as diet pills and laxatives, for weight control by adolescents is well-documented and can precipitate serious medical conditions. Yet only a small percentage of youth with disordered weight control behaviors receive treatment. The objective of this study was to examine how often clinicians communicate with youth with symptoms consistent with abuse of OTC products for weight control about possible use of these products.

We used electronic medical records and administrative claims for services for 53,229 12 to 17 year old patients receiving care from an integrated health system in the U.S. Northwest from August 2007 to December 2010. We examined electronic text of clinical notes to identify encounters in which the clinician noted one of 10 metabolic conditions potentially associated with abuse of OTC products (diet pills, laxatives, diuretics, ipecac, orlistat, and alli®) for weight control and then assessed whether clinicians noted communication with adolescent patients about possible use of OTC products for weight control.

**Results:**

We identified 130 (0.2% of sample) patients with clinical notes indicating one or more of the metabolic conditions. In clinical notes for only four (3.1%) of these patients did clinicians document suspicion or communication about possible abuse of the OTC products. All four had a previous eating disorder diagnosis. In the 12 months subsequent to the clinical encounter in which a metabolic disturbance was identified, medical notes for only three (2.3%) of the 130 patients indicated clinician suspicion or communication about possible abuse of these products or an eating disorder.

**Conclusions:**

Clinicians are missing a critical window of opportunity to query adolescents when presenting with suspicious metabolic disturbances about possible abuse of OTC products for weight control. Clinicians may need more training to detect OTC product abuse, and electronic medical records should prompt more thorough enquiry.

## Background

It is well-documented that adolescents abuse over-the-counter (OTC) products such as diet pills, laxatives, and diuretics in attempts to control their weight [1,2]. Estimates from representative samples suggest 5.9% of female and 4.2% of male U.S. high school students report using diet pills without a physician’s orders in the past month [2]. In a statewide representative sample of Minnesota high school students, 1.9% of females and 1.7% of males reported past-year laxative abuse for weight control [3]. Other studies with representative school-based samples of adolescents have found in both males and females that obese compared to nonoverweight youth reported higher past-week prevalence of use of diet pills and laxatives for weight control [4]; whereas, underweight males were more likely and underweight females were less likely than same-gender healthy weight youth to report use of diet pills, laxatives, or vomiting for weight control [5]. Lifetime prevalence of laxative abuse for weight control in the U.S. general population is estimated to be 4% [6].

Adverse effects associated with abuse of these products can be serious and even life threatening [7]. The consequences can include renal and hepatic failure, ischemic and hemorrhagic stroke, cardiac arrhythmia, hypokalemia, metabolic acidosis, and other electrolyte and fluid disorders [8-14]. Despite the known health risks, OTC diet pills, laxatives, and diuretics are widely available to youth in pharmacies and online with almost no restrictions [15]. Even when there are restrictions, as with orlistat, which is not approved for sale OTC to minors in the United States, access is fairly unfettered [15].

No studies we are aware of have examined clinician detection of OTC product abuse for weight control in primary care settings or possible bias, such as related to patient gender or weight status, in clinicians’ rate of detection. There has been some relevant research, however, on clinician detection practices and bias more broadly related to eating disorders (of which abuse of OTC products for weight control may be a symptom) and mental illness in general. The majority of youth with symptoms of eating disorders do not receive treatment [16,17], and underrecognition of eating disorder symptoms in clinical settings is well-documented [18,19]. Gender disparities in treatment access have been observed, where affected boys are substantially less likely than girls with comparable symptom severity to report receiving treatment [17], and primary care physicians are less likely to detect mental health problems in male than in female patients [20]. Assumptions that eating disorders primarily affect underweight individuals may also be a barrier to identification of symptoms and treatment access in overweight and healthy weight youth and adults [18,21,22].

Given that abuse of OTC products for weight control can precipitate serious metabolic disturbances requiring medical attention, the clinical encounter in which a youth presents with such a condition is a potentially important time to assess for OTC product use. Using a large medical claims database, we examined how often clinicians documented communicating with youth with metabolic disturbances consistent with abuse of OTC products for weight control about their possible use of these products. We hypothesized that clinicians would rarely communiate about abuse of these products with symptomatic patients. We further hypothesized that among patients with a metabolic disturbance, clinicians would be less likely to communicate with boys than girls and more likely to communicate with underweight than with other youth about abuse of OTC products for weight control.

In an additional examination, we investigated how often clinicians documented communicating with adolescent patients generally, regardless of evidence of metabolic disturbance, about use of OTC products for weight control. Among adolescent patients in general, we hypothesized that clinicians would be less likely to communicate with boys than girls and more likely to communicate with underweight than other youth about abuse of OTC products for weight control.

## Methods

Group Health Cooperative is an integrated health system in the U.S. Northwest serving approximately 640,000 members in Washington and Idaho. Group Health members are generally similar to the area population in distribution of age, sex, income, educational attainment, and race/ethnicity. Members are enrolled through a mixture of employer-sponsored insurance, individual insurance plans, Medicare, Medicaid, and other subsidized insurance programs for low-income residents. Group Health provides care through an internal group practice and a network of contracted external providers. All group-practice, outpatient facilities have used an electronic medical record system (EpicCare, Epic Systems, Verona, WI) since 2005. Our sample was restricted to enrollees seen by clinicians at integrated group-practice locations because contracted providers do not have access to the electronic medical record. This study was approved by the institutional review board of Group Health Cooperative.

We consulted with the research literature and expert clinicians to identify a list of 10 types of metabolic disturbances that may be associated with OTC products abused for weight control [7-13]. Using a combination of keyword searches of clinical text and International Classification of Diseases, Ninth Revision, Clinical Modification (ICD-9-CM) codes (automated claims data), we identified all instances of: electrolyte disturbance, hypochloremic alkalosis, hypermagnesmia, hypoalbuminemia, hypokalemia, hyperchloremic acidosis, hyponatremia, hypophospatemia, metabolic acidosis, and metabolic alkalosis.

We performed keyword searches of clinical text to identify suspected or confirmed use of OTC products for weight control. Our primary search terms included: diet pills, laxatives, diuretics, ipecac, orlistat, and alli®. Keywords were adapted using SAS “perl regular expressions” [23] to capture encounters with misspelled keywords. Each clinical note containing a reference to one or more of the 10 selected metabolic disturbances or potential use of weight loss products was reviewed individually. Potential cases with negating language (e.g., “patient denies use of diet pills, laxatives, or diuretics”) were coded as “suspected use.” Cases where clinicians noted endorsement of product use for weight control were coded as “confirmed use.”

Combined electronic medical records and administrative claims for services were used to identify all encounters possibly associated with abuse of OTC products for weight control. The full electronic text of clinical notes from ambulatory, emergency department, telephone, and secure electronic message encounters is downloaded from the electronic medical record to an indexed (searchable) research database daily. We used this database to search for encounters where the clinician noted or formally diagnosed one or more of the 10 selected metabolic disturbances (see list above). The keyword search of clinical notes produced a list of unique patient identification numbers. These identification numbers were used to create a database of all clinical notes and health care utilization (administrative claims) in the 12 months following the encounter at which a metabolic disturbance was documented.

For secondary analyses, we also selected all clinical notes of enrollees aged 12 to 17 years regardless of whether a metabolic disturbance was documented to have occurred. These notes were then flagged for any indication that the clinician had suspicions or communicated about OTC products for weight control.

### Sample

Our sample included all Group Health enrollees aged 12 to 17 years continuously enrolled for at least 10 months in any calendar year between Aug. 1, 2007, and Dec. 31, 2010. The study time frame corresponds to the earliest date that clinical notes were assembled and indexed in the research database. We excluded individuals from the metabolic-disturbance population if they had one or more medical conditions that would likely be associated with the disturbances. We used ICD-9-CM diagnosis codes to exclude anyone with a previous diagnosis of renal disease, mitochondrial disorders, muscular dystrophy, hypertension, or any cancer. These diseases were a basis for exclusion from our study because they and/or their treatment can cause metabolic disturbances similar to ones that can result from abuse of OTC products for weight control [24-27]. We did not exclude individuals with diabetes, as there is evidence that diabetic adolescents are at elevated risk for disordered weight control behaviors [28]. We did not exclude individuals with diagnoses related to acute viral infections or gastrointestinal problems; however, we did review notes individually for evidence of these conditions as the primary reason for the visit. Only one case needed to be eliminated due to the clinician’s assessment of gastroenteritis (noted as “viral syndrome”).

### Statistical analyses

Height and weight, recorded in the clinical note within 30 days prior and subsequent to the patient presenting with one or more of the selected metabolic disturbances, were used to calculate body mass index (BMI) percentile for age and sex following U.S. Centers for Disease Control and Prevention guidelines (http://www.cdc.gov/growthcharts/). Youth were then assigned weight status categories based on their BMI percentile as follows: overweight/obese: BMI ≥ 85th; healthy weight: 15th ⪯ BMI < 85th; underweight: BMI < 15th.

Among youth with one or more metabolic disturbance, Pearson Chi-square tests were used to examine for possible gender differences age, weight status, previous eating disorder diagnosis, and type of metabolic disturbance identified. While additional Chi-square tests had been planned to assess gender differences in clinician suspicion of OTC product use for weight control, patient denial of use, and patient endorsement of use, there were not sufficient cases to conduct statistical analyses of gender differences in these outcomes.

Among the sample of youth with a clinical note that included one of the OTC product terms included in our search, regardless of presence of a metabolic disturbance, we used Chi-square tests to examine for possible gender differences in clinician suspicion of OTC product use for weight control, patient denial of use, and patient endorsement of use, and previous eating disorder diagnosis. Pearson Chi-square tests were also used to examine for possible weight-status group differences in clinician communication about use of an OTC product for weight control. All analyses were performed with the statistical package SAS 9.2.

## Results

Our sample included 53,229 youth (26,015 females and 27,214 males) ages 12 to 17 years enrolled in Group Health Cooperative during the 29-month observation period. We identified 130 (0.2% of sample; 77 females and 53 males) patients with clinical notes indicating one or more of the selected metabolic disturbances. Based on 230 clinical notes from these 130 patients, Table 1 displays the sex, age, weight status, any eating disorder diagnosis, and type of metabolic disturbances noted for these patients. In medical notes for only four (3.1%) patients did the clinician document communication about possible abuse of the products during the encounter in which the disturbance was reported, and all four had a previous eating disorder diagnosis. In addition, in the 12 months subsequent to the clinical encounter in which a metabolic disturbance was noted, the medical notes for only three (2.3%) of the 130 patients documented that clinicians communicated about possible abuse of these products or an eating disorder.

**Table 1:** **Selected characteristics documented in 230 clinical notes for 130 adolescent patients (77 females and 53 males) ages 12-17 years presenting with a metabolic disturbance potentially associated with abuse of over-the-counter (OTC) products for weight control**^**a**^

	**Females**	**Males**	**P-Value**^**b**^
	**(N = 146 Notes)**	**(N = 84 Notes)**	
**Mean age, years (std. dev)**	15.5 (1.48)	15.5 (1.56)	0.8979
**Weight status**^**c**^**, % (n)**			
Underweight	16.9 (13)	22.6 (12)	0.049
Healthy weight	52.0 (40)	30.2 (16)	
Overweight	15.6 (12)	15.1 (8)	
Obese	15.6 (12)	32.1 (17)	
**Any previous eating disorder diagnosis, % (n)**	7.8 (6)	3.8 (2)	0.349
**Type of condition noted**^**d**^**, % (n)**			
Electrolyte disturbance	8.2 (12)	8.3 (7)	0.976
Hypochloremic alkalosis	0	0	N/A
Hypermagnesmia	0	0	N/A
Hypoalbuminemia	6.2 (9)	2.4 (2)	0.195
Hypokalemia	0	0	N/A
Hyperchloremic acidosis	0	0	N/A
Hyponatremia	0	0	N/A
Hypophospatemia	1.4 (2)	2.4 (2)	0.572
Metabolic acidosis	7.5 (11)	8.3 (7)	0.820
Metabolic alkalosis	4.8 (7)	3.6 (3)	0.661

^a^Data are aggregated from 230 notes over a 29-month observation period from Aug. 1, 2005, through Dec. 31, 2010.

^b^P-value from Pearson Chi-square test of gender differences.

^c^Weight status categories defined according to BMI percentile for age and sex as follows: obese: BMI ≥ 95th; overweight: 95th > BMI ≥ 85th; healthy weight: 15th ≤ BMI < 85th; underweight: BMI < 15th.

^d^Patients could be diagnosed with more than one metabolic disturbance.

In secondary analyses, results of our search for specific product terms (diet pills, laxatives, diuretics, ipecac, orlistat, and alli®), regardless of the presence or absence of a metabolic disturbance, are presented in Table 2. We identified 745 notes that included one or more of these search terms for 426 (0.8% of total sample) youth. By gender, we identified 588 notes for 307 females (1.2% of female sample) and 157 notes for 119 males (0.4% of male sample). In the 745 notes, clinicians documented suspicion of use of OTC products for weight control in 44 notes (5.9% of notes) and documented patient denial of use in 396 notes (53.2% of notes). Of the 426 youth, 12.4% (n = 53, all females) reported use of one or more of the products; almost 95% of admitted use was of laxatives and the remainder diet pills.

**Table 2:** **Selected characteristics documented in 745 clinical notes for 426 adolescent patients (307 females and 119 males) ages 12-17 years whose clinical note included mention of OTC products used for weight control**^**a**^

	**Females**	**Males**	**P-value**^**b**^
	**(N = 588 Notes)**	**(N = 157 Notes)**	
Mean age, years (std. dev.)	15.1 (1.6)	14.8 (1.6)	0.0031
Clinician notes suspicion of use of OTC products abused for weight control, % (n)	5.6 (33)	7.0 (11)	0.51
Patient denies use of OTC products abused for weight control (negating language used by clinician), % (n)	60.5 (356)	25.5 (40)	<0.0001
Patient endorses use, % (n)	9.0 (53)	0.0 (0)	<0.0001
Any previous eating disorder diagnosis, % (n patients)	29.0% (n = 89 cases/307 patients)	8.4% (n = 10 cases/119 patients)	<0.0001

^a^ Data are aggregated from 745 notes over a 29-month observation period from Aug. 1, 2005, through Dec. 31, 2010.

^b^ P-value from Pearson Chi-square test of gender differences.

Examining patterns by patient gender, we found that among the 426 youth with a note that included one of the OTC product terms used in our search, clinicians were more likely to document communicating with female than male patients, either by recording the patient endorsed use or denied use (P’s < 0.0001), though we did not find a difference by patient gender in clinicians noting suspicion of use (P = 0.51).

Table 3 shows patterns by adolescent weight status within the 745 clinical notes. We found that while more than 80% of notes for underweight youth indicated clinicians communicated with patients (either by recording the patient endorsed use or denied use) about use of OTC products for weight control, just over 40% of notes for overweight and obese youth indicated their clinicians communicated with them about use of these products (P < 0.0001 for difference across weight categories).

**Table 3:** **Documentation of clinician communication about use of OTC products for weight control in clinical notes by weight status for 426 adolescent patients (307 females and 119 males) ages 12-17 years (notes = 745, adolescents = 426)**^**a**^

			**P-value**^**b**^
**Weight status**^**c**^**, % (n)**	**Yes**	**No**	<0.0001
Underweight (notes = 273)	81.3 (222)	18.7 (51)	
Healthy weight (notes = 346)	63.0 (218)	37.0 (128)	
Overweight (notes = 71)	42.3 (30)	57.7 (41)	
Obese (notes = 55)	41.8 (23)	58.2 (32)	

^a^ Data are aggregated from 745 notes over a 29-month observation period from Aug. 1, 2005, through Dec. 31, 2010.

^b^ P-value from Pearson Chi-square test of weight-status group difference.

^c^ Weight status categories defined according to BMI percentile for age and sex as follows: obese: BMI ≥ 95th; overweight: 95th > BMI ≥ 85th; healthy weight: 15th ∩ BMI < 85th; underweight: BMI < 15th.

## Discussion

To our knowledge, ours is the first large-scale study to evaluate the frequency and patterns of clinicians communicating with youth about possible abuse of OTC products for weight control. Findings suggest clinicians rarely note suspicion or communication about the use of these products and likely are missing indicators of possible abuse of OTC products for weight control. Though self-report survey data from representative community-based samples estimate more than 5% of U.S. adolescents abuse OTC products for weight control each month, in our study of over 53,000 adolescent patients’ records, only 0.8% of patients’ notes documented suspicion or communication about these products. Even when patients presented with suspicious metabolic disturbances, such as hypokalemia and hyponatremia, very few notes indicated any clinician attention to possible OTC product abuse. Out of 130 youth presenting with suspicious metabolic disturbances, only four had records indicating the clinician communicated with the patient about possible abuse of OTC products for weight control at the time the disturbance was identified. All four of these adolescents had a previous eating disorder diagnosis, suggesting that the previous diagnosis, rather than the metabolic disturbance alone, might have prompted clinician attention.

Our study was in part designed to examine differences in clinician communication practices associated with patient gender and weight status among youth presenting with metabolic disturbances. Because so few patients with metabolic disturbances were queried, we were unable to conduct statistical tests to compare patterns within youth with these disturbances.

In secondary analyses, we identified youth whose clinical note included mention of OTC product terms used in our search, regardless of presence or absence of a metabolic disturbance. In support of our hypothesis about gender bias in clinician communication practices, we found a 3:1 (i.e., 1.2:0.4) female-to-male gender ratio in the prevalence of clinicians documenting communicating with patients regarding OTC products used for weight control. This gender ratio should be considered in light of results from representative, community-based research mentioned above, which estimates the female-to-male ratio in past-month diet pill use as approximately a 1.4:1 [2]. Our finding is consistent with prior research documenting primary care physician underrecognition of mental health problems in men compared to women patients [20]. In addition, our findings suggest clinician gender bias in communication practices may in part explain the results of another study documenting that when adolescent boys and girls with commensurate eating disorders symptom severity were compared, boys were much less likely than girls to have ever received treatment for their symptoms [17]. In contrast to our hypotheses, however, we found no difference by patient gender in clinician documentation of suspicion of use.

In addition, we found an inverse gradient of communication about OTC products associated with patient weight status, where the prevalence of documented clinician communication was highest among underweight youth and lowest among overweight and obese youth. This finding provides support for our hypothesis regarding weight-related bias in clinician communications and is consistent with prior research that eating disorder symptoms are underrecognized in clinical settings, especially among overweight and obese individuals who may be offered treatment for their weight but not for their underlying eating disorder symptoms [18,21,29].

Our study has several limitations. One, data were gathered from extensive electronic medical records and administrative claims for services; however, clinical notes may be incomplete because providers may not document all communications with patients for a variety of reasons, including time pressure in the clinical visit and concerns about confidentiality if providers believe parents or caregivers may have access to their minor child’s electronic records. Providers may do a complete review of systems, asking questions about weight control methods, yet document the communication only as a negative review of systems. As incomplete documentation has been a long-standing concern within healthcare systems generally [30-32], Group Health offers regular trainings for providers and conducts coding audits to improve quality and completeness of documentation of clinical encounters. Two, it was not possible to determine how many of the patients abused OTC products for weight control, as records capture only those whose clinicians documented both asking patients and the patients’ response. Furthermore, patients who use OTC products may deny use when asked in an effort to conceal stigmatized behavior [33]. Three, our search terms were deliberately inclusive with the goal of capturing all clinician notes related to OTC products used for weight control, yet it is possible some clinicians used different terms in their notes when communicating about these products. Four, while we found evidence to support our hypotheses regarding clinician bias in communication practices associated with patient gender and weight status, we were not able to examine the other possible types of clinical bias, such as bias related to patient race/ethnicity or socioeconomic position, as has been observed in other studies [34-37]. Five, we excluded patients who had been diagnosed with major illnesses in which the disease or its treatment may cause metabolic disturbances; however, in some cases, providers may have reasonably assumed the observed metabolic disturbances were explained by another known illness, such as gastroenteritis, rather than abuse of OTC products. That said, when we individually reviewed notes for patients with metabolic disturbances, we found only one case in which the clinician documented a gastroenteritis-like condition, specifically noting “viral syndrome.”

Survey research with U.S. adolescent girls and boys has well-documented abuse of OTC products for weight control. Representative, community-based research has estimated that more than 5% of youth report past-month use of OTC products for weight control, such as diet pills, laxatives, and diuretics [2]. Only a small percentage of youth with these and other eating disorder symptoms receive treatment [16,17], which may in part relate to our finding that clinicians rarely communicate about abuse of these products. Though abuse of OTC products for weight control can precipitate serious conditions requiring medical attention [7-14], they are widely and easily available to youth in pharmacies and online [15]. One important strategy proposed to reduce the harm these products pose to young people is to impose age restrictions on purchases [15]. Clinicians can also play an important role given that they are in a unique position to recognize signs and symptoms of OTC product abuse in patients and to help these youth adopt more healthful behaviors related to weight and shape.

## Conclusion

Clinical encounters in which a youth presents with a metabolic disturbance consistent with abuse of OTC products for weight control may be a critical window for clinicians to screen for this behavior and intervene if needed. Our findings suggest clinicians may need more training in recognizing symptoms of abuse of OTC products for weight control. In addition, the electronic medical record could be a useful tool in reminding clinicians to enquire about weight control products, especially for patients with eating disorder diagnoses or who present with an unexplained metabolic disturbance, by adding into the template prompts regarding communication on use of these products. It is worth noting that the majority of patients using these products may not present with substantial metabolic disturbance, as these problems are most likely to arise primarily in heavy users [9,10,38]. More thorough clinical enquiry is warranted about possible abuse of OTC products for weight control with both female and male adolescents as a whole across the weight-status spectrum in addition to those presenting with suspicious metabolic disturbances.

## Competing interests

The authors declare that they have no competing interests.

## Authors’ contributions

SBA, RBP, and JH conceptualized and designed the study. SBA, RBP, RLJ, JH, and SF planned data analyses, interpreted data, drafted the article, and revised it critically for important intellectual content. RBP and RLJ acquired the data and carried out analyses. Each author has given final approval of the version to be published. All persons who qualify for authorship are listed. Each author has participated sufficiently in the work to take public responsibility for appropriate portions of the content. All authors read and approved the final manuscript.
